# Internalized Stigma in Persons With Mental Illness in Qatar: A Cross-Sectional Study

**DOI:** 10.3389/fpubh.2021.685003

**Published:** 2021-06-07

**Authors:** Vahe Kehyayan, Ziyad Mahfoud, Suhaila Ghuloum, Tamara Marji, Hassen Al-Amin

**Affiliations:** ^1^Faculty of Nursing, University of Calgary in Qatar, Doha, Qatar; ^2^Department of Population Health Science, Weill Cornell Medicine - Qatar, Doha, Qatar; ^3^Mental Health Services, Hamad Medical Corporation, Department of Psychiatry, Weill Cornell Medicine - Qatar, Doha, Qatar; ^4^Department of Psychiatry, Weill Cornell Medicine - Qatar and Mental Health Services, Hamad Medical Corporation, Doha, Qatar

**Keywords:** stigma, internalized stigma, self-stigma, Qatar, mental illnes, internalized stigma mental illness scale

## Abstract

Stigma impacts persons with mental illness (PWMI), their families and network of friends, the public and health care professionals. Stigma is a major barrier for PWMI to seeking treatment, which contributes to the burden of disease, disability, and mortality. Research on stigma is relatively scant in the Middle East region and particularly in Qatar. To address stigma effectively in each culture, it is essential to study its nature in the context where the PWMI experience stigma. The purpose of this study was to assess the prevalence of internalized stigma in PWMI in Qatar. A cross-sectional study of PWMI receiving outpatient mental health services in Qatar was done. We interviewed 417 PWMI using a modified 18-item version of the short form of the Internalized Stigma of Mental Illness (ISMI) Scale. Descriptive and regression models were used to analyze the data. The Cronbach alpha for the modified 18-items ISMI was 0.87. Participants' average score on this scale was 2.07 ± 0.38 with 41 (9.8%) of them scoring more than 2.5 which is considered “high” stigma score. In multivariate logistic regression, high stigma (modified ISMI score >2.5) was significantly higher among PWMI with no formal education and among those who reported lower levels of social support. The reported levels of internalized stigma in this vulnerable population of Qatar fall at the lower spectrum reported worldwide. An anti-stigma education program designed for the context of Qatar emphasizing on education and support for PWMI may be conducive to creating an all-inclusive society.

## Introduction

Mental illness-related stigma and discrimination is widespread worldwide in the general public, health care professions, health service providers and policy makers, and even in persons with mental disorders (1–6). Several researchers have reported the existence of stigma in the Middle East (7–9), India (10), Europe (11), and the United States (12, 13) and elsewhere (14, 15). Stigma is a subjective and complex concept because individuals' experiences of stigma are influenced by their beliefs and culture (16). Studies have shown an association between experienced self-stigma and culture (7, 17, 18). Despite modern approaches to its treatment, mental illness is still attributed in some cultures to the supernatural, possession by spirits, magic, religion or punishment by God, including Arab cultures (17–20). A study that compared public attitudes toward auditory hallucinations in persons with mental illness reported more negative attitudes in Saudi Arabia than

in Britain. The former group attributed such symptoms to Satan, while those in Britain attributed them to schizophrenia or brain damage (21). In another study, medical students in Qatar had more negative attitudes toward mental illness than those in the State of New York (22).

Several studies conducted in Qatar exploring stigma related to mental illness had negative views of PWMI. In a large study involving 2,254 adults attending primary health care settings, almost 41.0% of the participants thought that PWMI were mentally retarded and that mental illness was a punishment from God (23). Another study of 2,514 adults attending primary health care settings in Qatar reported gender differences in how PWMI were perceived. Women, compared to men, had more negative views of mental illness. Women attributed mental illness to possession by evil spirits, were afraid talking to PWMI, and preferred traditional healers for treatment (24). Qatar is primarily a Muslim country. In Muslim cultures mental illness is attributed to supernatural interpretations (8, 20, 25). In this context, the PWMI is “considered to be possessed (meskoon) by supernatural beings or evil spirits (jinn) which control his/her behavior, thoughts, and desires” (18). Other Muslim communities view mental illness as a test from God or punishment for committed sins (8, 26). Such beliefs influence how mental illness is perceived in Qatar society and the experience of internalized stigma of PWMI (27–29).

Mental illness stigmatizes the individuals who have it and results in negative consequences, including discrimination (30). A global study involving 27 countries confirmed that persons with mental illness experienced and anticipated discrimination in many aspects of their lives, including making or keeping friends, finding employment, having intimate relations, and maintaining relations with family members (31). Anticipated discrimination compelled most of them not to reveal their mental disorder when seeking employment, education or close relationships (31). Furthermore, stigma is a major barrier to seeking treatment with the consequence that persons with mental illness (PWMI) do not receive necessary and timely treatment (32, 33). A systematic review of the literature from 1980 to 2011 involving 144 studies and over 90,000 subjects reported that “stigma was the fourth highest ranked barrier to help-seeking” (32). A global study of 17 countries reported that about two thirds of the population having mental disorders do not receive treatment (34). Untreated mental disorders have serious consequences. They are “associated with risk factors for chronic disease such as smoking, reduced activity, poor diet, obesity, and hypertension” (35). They also interact with and affect the rate of other health conditions such as cardiovascular diseases and diabetes (35). Stigma can also delay early recognition and intervention of mental disorders that are critical for successful recovery (36).

To address stigma effectively in a given culture, it is essential to study its nature in the context where the PWMI experience internalized stigma. To the knowledge of the researchers, such a study has not been done in Qatar. Therefore, the levels and perception of internalized stigma in PWMI in Qatar need to be studied and addressed. The objective of this present paper is to assess internalized stigma and its associated factors in PWMI. Given the societal and cultural context of the experience of internalized stigma, we hypothesized that the level of internalized stigma in PWMI in Qatar will be higher compared to those reported in published studies in European and North American countries.

## Methods

### Design, Participants, and Setting

This was a cross-sectional study of PWMI receiving outpatient mental health services at the Mental Health Service (MHS) in Doha, Qatar. It was one part of a mixed methods study exploring the perception of stigma in PWMI and in their identified families, and the attitude of healthcare professionals toward mental illness and PWMI.

The MHS is governed by the Hamad Medical Corporation (HMC): the principal provider of secondary and tertiary health care in Qatar. Data collection took place between May and October 2018. Potential male and female participants were included if they were 18 years of age and over; could speak Arabic, English or Urdu; could give informed consent as determined by a clinician; and gave signed, informed consent. Those with (1) a diagnosis of developmental disability, (2) a diagnosis of drug or alcohol addiction without having a diagnosis of mental disorder, or (3) who could not give informed consent as determined by a clinician were excluded from the study.

### Measures of Stigma

The *Internalized Stigma of Mental Illness (ISMI) Scale* was used to measure internalized stigma in persons with mental illness (37). The original ISMI Scale is a 29-item self-report questionnaire, grouped under five categories: alienation, stereotype endorsement, perceived discrimination, social withdrawal, and stigma resistance. The ISMI developer had subsequently designed a brief version consisting of only 10-items (38). The developers of ISMI had reported a Cronbach alpha of 0.92 for the 29-item ISMI and 0.75 for the 10 item ISMI with a Pearson's correlation of 0.94 between them (37). For the purposes of this study, the researchers opted to use the brief version with an additional eight items from the original scale for a total of 18-items (ISMI-18). The reason for this adaptation was to enable the researchers in the next phase of the study to compare the internalized stigma in PWMI with that in family members. The adaptation would allow comparison of internalized stigma between the two groups on 11 items of ISMI rather than only three. The Cronbach Alpha for the ISMI-18 was 0.865, while that for the 10-item version was 0.775. The correlation between ISMI-18 and ISMI-10 was *R* = 0.943 (*p* < 0.001). Moreover, participants' scores on ISMI-18 and ISMI-10 had almost identical means and standard deviations with no significant difference between them *p* > 0.05.

Each of the items in the adapted questionnaire is rated on a 4-point Likert scale as in the original 29-item ISMI: strongly disagree (1), disagree (2), agree (3), and strongly agree (4). Except for two items, higher scores on each of the items indicate higher levels of internalized or self-stigma. Those two items were reverse coded when scores were computed. A participant's score is computed by adding the scores of each answered item and dividing it by the number of answered items. The resulting score ranges from 1 to 4. The scores are interpreted as follows: 1.00 to 2.00 = minimal to no internalized stigma; 2.01 to 2.50 = mild internalized stigma; 2.51 to 3.00 = moderate internalized stigma; and 3.01 to 4.00 = severe internalized stigma (39). Using the original 29 item ISMI as a reference, questions were grouped to form 5 subscales: alienation (based on four questions), stereotype endorsement (based on five questions), discrimination (based on four questions), social withdrawal (based on three questions), and resistance (based on two questions, the same two questions whose scores were reverse coded in the computation of the 18-item scale score). Reliability measures of the subscales using Cronbach's alpha were 0.46, 0.55, 0.58, 0.70, and 0.77 for resistance, social withdrawal, alienation, stereotype endorsement and discrimination respectively. Participants' scores on the subscales were computed in a similar manner described for the overall 18-item ISMI. Scores on the subscales would range from 1 to 4 with higher values indicating more stigma in the subscales except for the resistance subscale where higher numbers indicate higher resistance.

### Translation of ISMI

The study participants' spoken languages were Arabic, English or Urdu. While the ISMI 29-item instrument has been translated into 60 languages, including Arabic and Urdu (J. E. Boyd, personal communication, December 9, 2018), we translated the English version of the 18-item scale into Arabic and Urdu using forward and backward translation methodology to contextualize ISMI for Qatar's cultural context. In addition, both translations were critically reviewed and verified academicians who were proficient in these two languages. The back translations were compared with the original English version and discrepancies were discussed with the ISMI developer, resulting in further editing to the translations as needed.

### Data Collection Procedure

The participants' mental disorder diagnoses and mental illness related information; such as the duration of mental illness, number of hospitalization due to mental illness, number and type of interventions they received as treatment for mental illness, and their level of insight about their disease were retrieved from their electronic health records. Most psychiatrists in the MHS follow DSM 4/5 (Diagnostic and Statistical Manual of Mental Disorders) or ICD-10 (International Classification of Diseases) criteria for mental disorder diagnoses.

The 18-item ISMI Scale was administered by three Research Assistants (RAs). The lead researcher trained them in all aspects of the research, including recruiting participants, conducting interviews and entering data. Potential study participants were screened by clinic staff and referred them to the RAs. The RAs explained; using the preferred language of the participant, the nature and purpose of the study using a prepared script and obtained the participant's signed consents. The RAs conducted the interviews in a structured-interview format in the participants' language of choice (English, Arabic, or Urdu). The RAs conducted the interviews by reading out each of the 18-items to the participants and recording their responses. This option of “reading” the questions by the RAs is consistent with other research (13). To assist the participants in their responses, the RAs provided them with a paper copy of the ISMI scale's four response options.

In addition, the RAs collected the following participants' demographic characteristics: age, gender, marital status, ethnicity, education level, work status, income source, income, religion, living status, and perceived level of social support. No intrusive procedures, drugs, or medical or diagnostic procedures were used following the one-time interviews described above. Consistent with other research (13) and with the approval of the ethics boards, an incentive payment was offered to the participants in the form of a gift certificate valued at 50.00 Qatari Riyals (QR; ~US $ 13.74) for participation. Following the interviews, the RAs entered the data into SurveyGizmo that is a web-based online survey platform.

### Data Quality Management

A pilot study was conducted with four Urdu, five English, and seven Arabic participants to ensure the feasibility of the study procedures, the comprehension of the language in the data collection forms, and the cultural validation of the 18-item ISMI Scale. No adjustments or revisions were deemed necessary. Additionally, ten per cent (10%) of all data entered into SurveyGizmo was selected at random, and two RAs verified data entry against the completed surveys. No discrepancies were found.

### Data Management, Confidentiality, and Anonymity

SurveyGizmo generated Unique Study Identification Numbers (USID) to each electronic form to ensure participants' anonymity and confidentiality. A separate register was kept by the RAs linking USIDs to participants' health card numbers to enable their electronic health records to be accessed for mental health related data. The lead researcher kept this record in secure storage. The register allowed for retrieval of completed surveys in case any of the participants wished to withdraw from the study. No participants request to withdraw from the study.

### Ethical Approvals and Consent

Ethics clearances were obtained from the Institutional Review Boards (IRB) of Hamad Medical Corporation, Weill Cornell Medicine in Qatar, and the University of Calgary in Alberta, Canada. Written informed consents were collected from all participants in the study.

### Sampling and Sample Size Calculation

A convenience sampling procedure was used in this study as described above. With 385 participants, the study was projected to estimate the mean value of the stigma scale to within 10% of the corresponding standard deviation using a 95% confidence interval. Moreover, this sample size was expected to allow the effects of at least 10 covariates on the value of the scale to be looked at using linear regression (40). The sample size was also considered appropriate for estimating the prevalence of participants with high stigma to within at most 5% margin of error using 95% confidence intervals.

### Statistical Analysis

All data in SurveyGizmo was uploaded into the IBM-SPSS (version 23) software for data analyses. Demographic and clinical characteristics were summarized using frequency distributions. The scores on the 18-item ISMI scales and those for the subscales were computed for each participant and were summarized using means and standard deviations (SD). Bivariate association between 18- item ISMI scores and each of the demographic and clinical characteristics were assessed using simple linear regression. Variables that were significant at the bivariate level were used in multivariate linear regression analysis. Adjusted slopes (regression coefficients) along with their corresponding 95% confidence intervals and *p*-values were calculated. High stigma was computed as someone having an a score larger than 2.5 on the 18-item ISMI scale (37). The association between high stigma and demographic and clinical characteristics was assessed using bivariate followed by multivariate logistic regression models. Variables that were significant at the bivariate level were included in the final multivariate model. Statistical significance was set at the 5% level. Except for computing the 18-Item ISMI score, listwise deletion was used for missing data in bivariate and multivariate analyses described above.

## Results

### Sociodemographic Characteristics

A total of 537 PWMI were asked to participate, 423 (79%) accepted to participate and 417 were analyzed since 6 patients did not complete the 18-item ISMI scale. Main reasons for refusal to participate were “don't have time or in a hurry” 41%, “no given reason” 20%, “not comfortable participating, or taking decision to participate,” 14%, currently tired or not in the mood” 10.0%, “denied having mental health” 5% and “not interested in research” 4%.

Detailed sociodemographic and clinical characteristics are presented in Table 1. A total of 417 PWMI were interviewed consisting of 206 females (49.40%) and 211 males (50.60%). Over 55% were 36 years of age and older and 65.23 % (*n* = 272) were married/engaged. Slightly over 52% (*n* = 217) were Arabs with Qatari nationals making up 14.39% (*n* = 60). The majority (66.92%; *n* = 279) had higher than secondary education, 56.34% (*n* = 231) were working, 36.39% (147) had no income, 16.34% (*n* = 66) had monthly incomes of less than US $1,375 (5,000 QR), while almost 31% (*n* = 124) had incomes of 10,000 QR or more, 76.74% (*n* = 320) were Muslim, 77.40% (*n* = 322) lived with friends or co-workers, and almost 70% (*n* = 289) reported to have good level of social support.

**Table 1:** Patients' socio-demographic and clinical characteristics (*N* = 417).

	***N* (%)**
**Age**	
18–25	59 (14.18%)
26–30	58 (13.94%)
31–35	69 (16.59%)
36–40	78 (18.75%)
41–50	94 (22.60%)
>60	58 (13.94%)
**Gender**	
Male	211 (50.60%)
Female	206 (49.40%)
**Marital status**	
Single	126 (30.22%)
Married/engaged	272 (65.23%)
Divorced/widowed	19 (4.56%)
**Ethnicity**	
Qatar	60 (14.39%)
Other Arab	157 (37.65%)
South Asia	121 (29.02%)
Others	79 (18.94%)
**Education level**	
No formal education	11 (2.64%)
Primary/Intermediate	47 (11.27%)
Secondary	80 (19.18%)
Some College or Technical/College or University degrees	279 (66.91%)
**Income**	
None	63 (15.14%)
Work	247 (59.38%)
Retired/Family support/Government	106 (25.48%)
**Work**	
Currently working	231 (56.34%)
Currently not working	179 (43.66%)
**Income**	
No Income	147 (36.39%)
<5000 QR^a^	66 (16.34%)
5,000–9,999	67 (16.58%)
>10,000 QR	124 (30.69%)
**Religion**	
Islam	320 (76.74%)
Christianity	65 (15.59%)
Hindu/Other	25 (6.00%)
Atheist/Not declared	7 (1.68%)
**Living**	
Alone	42 (10.10%)
With family	322 (77.40%)
With friends or co-worker	52 (12.50%)
**Level of social support**	
Good	289 (69.47%)
Fair	88 (21.15%)
Poor	39 (9.38%)
**Diagnosis**	
Not recorded	11 (2.66%)
Unipolar depression	154 (37.20%)
Bipolar disorder	59 (14.25%)
Anxiety and OCD^b^	118 (28.50%)
Psychotic disorders	57 (13.77%)
Others (Insomnia, personality disorder, ADHD^c^)	15 (3.62%)
**Average duration of mental disorder (in years)**	
<1	66 (16.18%)
1–2.9	150 (36.76%)
3–4.9	45 (11.03%)
5–6.9	30 (7.35%)
7–10	34 (8.33%)
>10	83 (20.34%)
**Level of insight**	
No	7 (1.72%)
Partial	11 (2.70%)
Full	389 (95.58%)
**Number of hospital admissions due to mental illness**	
<2	368 (90.20%)
2–5	31 (7.60%)
>5	9 (2.21%)
No prior inpatient admissions	0 (0.00%)
**Intervention**	
No medication	18 (4.35%)
Only medication	270 (65.22%)
Medication and other therapies	126 (30.43%)

a *QR, Qatari Riyal.*

b *OCD, Obsessive compulsive disorder.*

c *ADHD, Attention-Deficit/Hyperactivity Disorder*.

### Clinical Characteristics

Clinical characteristics are also presented in Table 1. The most prevalent mental disorder was unipolar depression (37.20%; *n* = 154); 42.75% (*n* = 177) had bipolar disorder, anxiety and/or obsessive-compulsive disorder, while 13.77% (*n* = 57) had psychotic disorders. About 53% (*n* = 216) had a duration of their mental illness under 3 years, while almost one fifth had their illness for more than 10 years, majority (95.58%) had insight (that is, were aware of their illness), 90.20% had <2 previous hospital admissions due to mental illness, and 65.22% received only medication for their therapeutic intervention.

### Levels of Self-Stigma

The score for the 18-items ISMI scale were approximately normally distributed (as per the use of histogram and Q-Q plots for the distribution of the scores) and ranged from 1.00 to 3.11 with an average of 2.07 and a standard deviation (SD) of 0.38. There were 41 (9.8% with 95%CI: 7.1%-13.1%) participants who were characterized as having “high” stigma score, which is a score of more than 2.5 on the 18 item ISMI scale.

Table 2 presents the frequency distribution of high stigma scores and its association with respect to participants' sociodemographic and clinical characteristics. Those with high levels of education and good social support were significantly less likely to report high stigma scores. In particular, as compared to those with good social support, those with poor social support had 5 times the odds of reporting high stigma scores (OR = 5.19 with 95% CI: 2.19–12.30).

**Table 2:** Association between high stigma (>2.5) scores and patients' characteristics.

	**High stigma**			
	***N***	**%**	***P*-value**	**OR [95% CI]**
**Age**				
18–25	8	13.6%		1.00
26–30	6	10.3%	0.593	0.74 [0.24, 2.27]
31–35	6	8.7%	0.383	0.61 [0.20, 1.86]
36–40	7	9.0%	0.398	0.63 [0.21, 1.84]
41–50	9	9.6%	0.447	0.68 [0.24, 1.86]
Above 60	5	8.6%	0.399	0.60 [0.18, 1.96]
**Gender**				
Male	24	11.4%		1.00
Female	17	8.3%	0.286	0.70 [0.36, 1.35]
**Marital status**				
Single	17	13.5%		1.00
Married/engaged	22	8.1%	0.095	0.56 [0.29, 1.10]
Divorced/widowed	2	10.5%	0.722	0.75 [0.16, 3.56]
**Ethnicity**				
Qatar	7	11.7%		1.00
Other Arab	13	8.3%	0.443	0.68 [0.26, 1.81]
South Asia	18	14.9%	0.557	1.32 [0.52, 3.37]
Others	3	3.8%	0.090	0.30 [0.07, 1.21]
**Education level**				
No formal education	3	27.3%		1.00
Primary/intermediate	8	17.0%	0.439	0.55 [0.12, 2.52]
Secondary	9	11.3%	0.156	0.34 [0.08, 1.51]
Some college or technical/college or university digress	21	7.5%	0.032^*^	0.22 [0.05, 0.88]
**Income source**				
None	10	15.9%		1.00
Work	21	8.5%	0.087	0.49 [0.22, 1.11]
Retired/family support/government	10	9.4%	0.215	0.55 [0.22, 1.41]
**Work**				
Currently working	20	8.7%		1.00
Currently not working	20	11.2%	0.396	1.33[0.69, 2.55]
**Income**				
No income	17	11.6%		1.00
<5,000 QR^a^	9	13.6%	0.670	1.21 [0.51, 2.87]
5,000–9,999	8	11.9%	0.937	1.04 [0.42, 2.54]
>10,000 QR	7	5.6%	0.094	0.46 [0.18, 1.14]
**Religion**				
Islam	36	11.3%		1.00
Christianity	3	4.6%	0.119	0.38 [0.11, 1.28]
Hindu/other	2	8.0%	0.619	0.69 [0.16, 3.03]
Atheist/not declared	0	0.0%	0.999	0.00 [0.00,]
**Living**				
Alone	6	14.3%		1.00
With family	26	8.1%	0.188	0.53 [0.20, 1.37]
With friends or co-worker	9	17.3%	0.691	1.26 [0.41, 3.86]
**Level of social support**				
Good	18	6.2%		1.00
Fair	12	13.6%	0.028^*^	2.38 [1.10, 5.15]
Poor	10	25.6%	0.001^*^	5.19 [2.19, 12.30]
**Diagnosis**				
Not recorded	2	18.2%		1.00
Unipolar depression	10	6.5%	0.170	0.31 [0.06, 1.64]
Bipolar disorder	8	13.6%	0.689	0.71 [0.13, 3.88]
Anxiety and OCD^b^	11	9.3%	0.361	0.46 [0.09, 2.42]
Psychotic disorders	6	10.5%	0.476	0.53 [0.09, 3.05]
Others (insomnia, personality disorder, ADHD^c^)	2	13.3%	0.736	0.69 [0.08, 5.86]
**Average duration of mental disorder (in years)**				
<1	6	9.1%		1.00
1–2.9	11	7.3%	0.659	0.79 [0.28, 2.24]
3–4.9	4	8.9%	0.971	0.98 [0.26, 3.67]
5–6.9	5	16.7%	0.287	2.00 [0.56, 7.16]
7–10	5	14.7%	0.399	1.72 [0.49, 6.12]
>10	6	7.2%	0.679	0.78 [0.24, 2.54]
**Level of insight**				
No	2	28.6%		1.00
Partial	2	18.2%	0.608	0.56 [0.06, 5.24]
Full	33	8.5%	0.088	0.23 [0.04, 1.24]
**Number of hospital admissions due to mental illness**				
<2	31	8.4%	0.267	1.00
2–5	4	12.9%	0.401	1.61 [0.53, 4.90]
<5	2	22.2%	0.169	3.11 [0.62, 1.24]
No prior inpatient admissions				
**Intervention**				
No medication	3	16.7%		1.00
Only medication	24	8.9%	0.282	0.49 [0.13, 1.81]
Medication and other therapies	12	9.5%	0.360	0.53 [0.13, 2.08]

a *QR, Qatari Riyal.*

b *OCD, Obsessive compulsive disorder.*

c *ADHD, Attention-Deficit/Hyperactivity Disorder; Anxiety disorders do not include post traumatic or other trauma-associated disorders*

* *significant difference p <0.05*.

Table 3 shows summary statistics for the participants' scores on the sub-scales along with the percentage of them with high scores (defined as a score of more than 2.5) within each subscale. However, as we used only a modified 18-items version of the 10-item ISMI scale, our results on subscales are only exploratory in nature and to be replicated in future studies. As shown, alienation had the highest mean score of 2.24 (SD = 0.5) followed by social withdrawal score of 2.22 (SD = 0.52). The stigma resistance score was 3.12 (SD = 0.51), and recoded (that is reversed) stigma resistance sub-scale, which we used for our overall mean score calculation, was 1.88 (SD = 0.51). High stigma resistance scores were observed in more than 80% of the participants. Moreover, there were significant positive correlations between the 18-item score and the scores on the stereotype endorsement subscale (r = 0.83), discrimination subscale (r = 0.81), alienation subscale (r = 0.79) and the social withdrawal subscale (r = 0.75) and significant negative correlations with the resistance subscale (r = −0.54, p < 0.01) indicating the higher the resistance scores the lower the stigma scores (data not shown).

**Table 3:** Distribution of scores on the 18-item ISMI and sub-scales.

						**Score** **≤** **2.5**		**Score** **>** **2.5**	
	**2**	S.DQ24.^**^	**Median**	**Min**	**Max**	***N***	**%**	***N***	**%**
18-item ISMI	2.07	0.38	2.11	1.00	3.11	376	90.20	41	9.80
Alienation (4 vs. 6)^*^	2.24	0.50	2.25	1.00	4.00	335	80.30	82	19.70
Stereotype endorsement (5 vs. 7)^*^	1.97	0.45	2.00	1.00	3.20	375	89.90	42	10.10
Discrimination (4 vs. 5)^*^	2.03	0.50	2.00	1.00	3.50	370	89.60	43	10.40
Social withdrawal (3 vs. 6)^*^	2.22	0.52	2.33	1.00	4.00	314	75.80	100	24.20
Resistance (2 vs. 5)^*^	3.12	0.51	3.00	1.00	4.00	69	16.5	348	83.5
Resistance recode	1.88	0.51	2.00	1.00	4.00	402	96.40	15	3.60

* *In the original 29-item ISMI scale, the subscales had larger number of items than the 18-item one we used in this study. For example, in the original 29-item ISMI, the “alienation” subscale had six items, in ours it had four.*

** *SD, Standard deviation.*

### Correlates of Self-Stigma

Table 4 presents the results of bivariate analyses between the scores on the 18-item ISMI scale and participants' characteristics. Those scores were significantly associated with participants' ethnicity with South Asians having the highest mean score (p < 0.001), level of education with those having no formal education having the highest mean score (p < 0.001), level of income with those having an income of less than 5000 QR (p <0.001), religion with Muslims having the highest mean score (p = 0.0282), those living with friends or co-workers having the highest mean score (p = 0.0033), those with poor level of social support (p < 0.001), and with psychotic disorders and other diagnoses having the highest mean score (p = 0.0114).

**Table 4:** Association between mean stigma scores and patients' characteristics.

**Characteristics**	**Mean**	**SD^a^**	**p-value**
**Age current**			
18–25	2.11	0.37	0.903
26–30	2.09	0.38	
31–35	2.08	0.35	
36–40	2.06	0.37	
41–50	2.08	0.40	
>60	2.03	0.39	
**Gender**			
Male	2.09	0.39	0.456
Female	2.06	0.37	
Marital status
Single	2.12	0.38	0.280
Married/engaged	2.05	0.38	
Divorced/widowed	2.09	0.34	
**Ethnicity**			
Qatar	2.11	0.36	<0.001
Other Arab	2.03	0.39	
South Asia	2.19	0.34	
Others	1.95	0.37	
**Education level**			
No formal education	2.41	0.26	<0.001
Primary/intermediate	2.23	0.30	
Secondary	2.17	0.29	
Some college or technical/college or university degrees	2.01	0.39	
**Income source**			
None	2.08	0.43	0.560
Work	2.06	0.39	
Retired/family support/government	2.10	0.32	
**Work**			
Currently working	2.05	0.39	0.130
Currently not working	2.11	0.36	
**Income**			
No income	2.12	0.36	<0.001
<5,000 QR^b^	2.20	0.36	
5,000–9,999	2.12	0.29	
>10,000 QR^b^	1.95	0.42	
**Religion**			
Islam	2.10	0.38	0.0282
Christian	1.99	0.35	
Hindu/other	2.02	0.35	
Atheist/not declared	1.81	0.36	
**Living**			
Alone	2.02	0.40	0.0033
With family	2.05	0.37	
With friends or co-worker	2.24	0.35	
**Level of social support**			
Good	2.02	0.37	<0.001
Fair	2.18	0.33	
Poor	2.23	0.42	
**Diagnosis category**			
Not recorded	2.25	0.28	0.0114
Unipolar depression	2.04	0.38	
Bipolar disorder	2.09	0.42	
Anxiety and OCD^c^	2.00	0.37	
Psychotic disorders	2.19	0.32	
Others (insomnia, personality disorder, ADHD^d^)	2.19	0.30	
**Average duration of mental illness (in years)**			
<1	2.17	0.29	0.129
1–2.9	2.03	0.39	
3–4.9	2.09	0.29	
5–6.9	2.10	0.42	
7–10	2.08	0.44	
>10	2.03	0.39	
**Level of insight**			
No	2.24	0.42	0.273
Partial	2.18	0.33	
Full	2.06	0.37	
**Number of hospital admissions due to mental illness**			
<2	2.06	0.38	0.264
2–5	2.17	0.36	
>5	2.12	0.36	
**Intervention**			
No medication	2.18	0.35	0.384
Only medication	2.06	0.38	
Medication and other therapies	2.08	0.37	

a *SD, Standard deviation.*

b *QR, Qatari Riyal.*

c *OCD, Obsessive compulsive disorder.*

d *ADHD, Attention-Deficit/Hyperactivity Disorder*.

In multivariate linear regression model (Table 5) those with more than secondary education had significantly lower stigma scores than those with no formal education (adjusted slope = −0.248; p = 0.029). Participants with reported income of 10,000 QR (US 2,746) or more also had significantly lower stigma scores than those with no income (adjusted slope = −0.113; p = 0.016). Those living with friends or co-workers had significantly higher stigma scores as compared to those living alone (adjusted mean difference = 0.167; p = 0.036) and those who had poor or fair social support reported higher stigma scores than those with good social support (adjusted mean difference = 0.235 with p < 0.001 for poor and 0.147 with p = 0.001 for fair). The adjusted R^2^ was 0.1593; that is, the model explains about 16% of the variation in ISMI scores.

**Table 5:** Multiple linear regression analysis of ISMI scores.

**Characteristics**	**Regression coefficient**	**SE^a^**	***P* > *t* [95% CI^b^]**
**Ethnicity**			
Other Arab	−0.099	0.058	0.087 [−0.212–0.014]
South Asia	0.014	0.061	0.821 [−0.107–0.135]
Others	−0.100	0.073	0.170 [−0.243–0.043]
**Education**			
Primary/intermediate	−0.160	0.119	0.177 [−0.393–0.073]
Secondary	−0.150	0.115	0.192 [−0.377–0.076]
Some college or technical/college or university degrees	−0.248	0.113	0.029 [−0.471–−0.025]
**Income**			
<5,000 QR^c^	0.002	0.064	0.970 [−0.123–0.128]
5,000–9,999	0.005	0.055	0.921 [−0.103–0.114]
>10,000 QR^c^	−0.113	0.047	0.016 [−0.206–−0.021]
**Religion**			
Christian	0.002	0.061	0.974 [−0.118–0.122]
Hindu/other	−0.083	0.083	0.314 [−0.245–0.079]
Atheist/not declared	−0.158	0.138	0.252 [−0.43–0.113]
**Living**			
With family	0.046	0.064	0.475 [−0.08–0.172]
With friends or co-worker	0.167	0.079	0.036 [0.011–0.322]
**Social support**			
Fair	0.147	0.044	0.001 [0.06–0.234]
Poor	0.235	0.064	0.000 [0.109–0.361]
**Diagnosis**			
Unipolar depression	−0.108	0.11	0.326 [−0.325–0.108]
Bipolar disorder	−0.086	0.116	0.460 [−0.314–0.142]
Anxiety and OCD^d^	−0.174	0.111	0.117 [−0.393–0.044]
Psychotic disorders	−0.030	0.116	0.796 [−0.258–0.198]
Others (insomnia, personality disorder, ADHD^e^)	−0.038	0.142	0.787 [−0.317–0.241]

a *SE, Standard error.*

b *CI, Confidence interval.*

c *QR, Qatari riyal.*

d *OCD, Obsessive compulsive disorder.*

e *ADHD, Attention-Deficit/Hyperactivity Disorder (ADHD)*.

In multivariate logistic regression model (Table 6), high stigma was significantly associated with the participants' educational levels and levels of social support. Participants with some level of education were less likely to report high stigma scores compared to no formal education. Also, the lower the level of social support, the higher the odds of high stigma. In particular, participants with poor social support had significantly higher odds of reporting high stigma compared to participants with good social support (OR = 5.54, *p* < 0.001).

**Table 6:** Multiple logistic regression analysis of ISMI scores.

	***p*-value**	**OR^a^ [95% CI^b^]**
**Education level**		
No formal education	0.045	1
Primary/intermediate	0.390	0.502 [0.104, 2.418]
Secondary	0.136	0.311 [0.067, 1.444]
Some college or technical/college or university degrees	0.025	0.191 [0.045, 0.811]
**Level of social support**		
Good	0.001	1
Fair	0.060	2.136 [0.969, 4.709]
Poor	0.000	5.538 [2.302, 13.322]

a *OR, Odds ratio.*

b *CI, Confidence intervals*.

## Discussion

The purpose of this study was to examine the levels and perception of internalized stigma in PWMI who attend outpatient mental health service clinics in Qatar. To our knowledge, this is the first study in Qatar about internalized stigma in persons diagnosed with a mental disorder.

In line with previous research (11–14), this study found that PWMI experienced internalized stigma albeit at different levels. The self-reported internalized stigma in this study population, using a modified 18-item ISMI scale, had a range of 1.00 to 3.11 with a mean overall score of 2.074 (SD 0.377). A mean score of 2.074 is considered mild according to the ISMI instrument (37). Despite the mild category of this mean score, most participants self-reported a certain level of internalized stigma. This supports the findings of other studies that internalized stigma is prevalent in persons with mental illness in many cultures. For instance, in a Pan European study involving six countries, an overall mean score of 2.3 (SD 0.5) was reported (2). In a community-based study in the United States, a mean total score of 1.29 was reported (SD 0.57) (13). In a study in Maryland, United States, the mean total ISMI score was 2.3 [SD ± 0.4] (12). In The GAMIAN-Europe study of 13 countries, varying levels of mean stigma between countries were found ranging from 1.61 (SD = 0.45) in Sweden to 2.36 (SD 0.40) in Lithuania, and a midpoint mean score of 2.22 (SD = 0.57) in Macedonia (41). In the studies reported, either the 10-item or the 29-item ISMI was used as the measure of internalized stigma.

A small percentage (9.80%) of the participants reported high stigma scores (>2.5). This is substantially lower than those reported by Boyd et al. (42) in their multinational review of ISMI scores including European, Asian, North American, and Middle Eastern countries (42). Almost one-third to one-quarter of the participants in those studies had reported high stigma. With a few exceptions, the percentage of high stigma scores in each of the sub-scales was lower than those reported by Boyd et al. (42). This may be due to differences in sample populations (e.g., forensic patients; patients with epilepsy) and data collection methods (e.g., postal survey vs face-to-face interviews).

In our study we had hypothesized that internalized stigma in the study population will be higher in Qatar, given its cultural and religious context (43, 44), compared to stigma levels in European or North American countries. In some instances, our hypothesis was held true, while in others it did not. We compared our 18-item ISMI score with studies that had also used the 29 item ISMI instrument. For instance, our mean score of 2.074 (SD 0.377; range 1.00 – 3.11) is slightly lower than Krajewski et al. (2) Pan European study with a mean score of 2.3 (SD 0.5) and Drapalski et al. (12) 2.3 (SD 0.4) score. However, our 18-item score was higher than Sweden's 1.61 (SD 0.45) (41), and West et al. (13) study in the United States of 1.29 (SD 0.57) (13).

### Predictors/Correlates of Stigma

In this study, we explored if any of the participants' socio-demographic characteristics might potentially predict or correlate with internalized stigma. We found that internalized stigma was not significantly associated with several sociodemographic characteristics, such as age, gender, marital or employment status, and source of income. This finding agrees with other studies (e.g., 12). However, Krajewski et al. (2) reported that in unadjusted general linear models, the mean ISMI score was significantly higher in men than in women: 2.4 (95% CI = 2.3–2.4) vs. 2.2 (95% CI = 2.2–2.3) respectively (2). While in our study, employment was not significantly associated with ISMI, Evans-Lacko et al. (11) reported such an association (11). Our finding of education being significantly associated with stigma levels agrees with other studies (2, 11). Our finding of significant association of level of social support with ISMI score was also reported in other studies (14).

We also explored if internalized stigma was associated with the participants' clinical characteristics. Our finding of a significant association between their psychiatric diagnoses and stigma scores (*p* = 0.0114) was similar to other studies (2, 9) that also reported such an association. Possible mediating effects of taking psychotropic medications or talking to someone about mental illness on perceptions of internalized stigma would have been interesting to explore in our study, as others had done (11). Level of insight (as determined by their clinician), number of hospitalizations due to mental illness, average duration of mental illness, and type of intervention received (e.g., medications, therapies) were not statistically significant in bivariate analyses, while other studies reported such associations (9, 14).

### Implications to Practice, Research, and Policy

The findings of this study have potential implications to practice. Clinicians should be mindful of their clients' experience of internalized stigma and discuss this phenomenon with them and the negative impact it may have on their journey to recovery from mental illness. Evans-Lacko et al. (11) reported in their study of stigma in 14 European countries involving 1,835 persons with mental illness that talking to someone about mental illness decreased self-stigma scores (11). While in our study we did not capture such data, it may be a relevant factor and may explain the mild stigma score given that the participants were actively receiving mental health services in the MHS outpatient program. We contend that the participants in our study were experiencing lower stigma because they were seeking mental health services as several studies have reported that the experience of self-stigma is a barrier to help seeking (32, 33). Furthermore, a large cross-sectional study in Finland compared the attitude of users of mental health services with non-users toward mental illness (45). The researchers reported that users had more positive attitude toward mental health than non-users. Hence, if PWMI are receiving mental health services, then they may be coping well with their mental illness and associated stigma. Another explanation for the mild overall mean score may be that the overall mean score for the stigma resistance sub-scale in our study was 3.12 (SD = 0.51) higher than the mean scores for the other subscales. Interestingly in a similar study in Ethiopia using ISMI, the stigma resistance score was 2.20 (SD = 0.34) much lower than ours. Finally, one other possible explanation for the low average ISMI score may be attributed to the ISMI scale itself influencing the choice of answers of the participants. For example, one of the ISMI items states “mentally ill people tend to be violent” and another one states “mentally ill people shouldn't get married”. In our study, over 65% of the participants were married. While the average ISMI score was mild, in our logistic regression model where we used 2.51 as the cut-off for high stigma (46), high stigma was experienced by 9.80% of the participants. Also, in the qualitative arm of this study all participants, except for one, took stringent measures not to disclose their mental illness to their network of friends and employers fearing discrimination and stigmatization (47).

Our study has implications for future research. For one, it would be interesting to examine, in the Qatari context, the experience of internalized stigma in PWMI in those who are not users of mental health services. Another possible future study could be again studying internalized stigma in this vulnerable population using the full ISMI scale.

### Strengths and Limitations

This study had several strengths related to the data we gathered. For one, the large sample size (*N* = 417) allowed for obtaining good precision with the computed estimates. Secondly, we adopted very stringent protocols and quality assurance procedures for the conduct of this study.

We also had a number of limitations that need to be mentioned. The cross-sectional nature of the study prevents us from drawing causality conclusions. Convenience sampling limits generalization of the findings to outpatient clients in Qatar. We used a modified version of the ISMI scale for the purposes of the study without subjecting it to psychometric testing although we obtained the approval from the ISMI developer. Still, our Cronbach alpha of 0.865 is reasonable and the scores on the 18-item ISMI had high correlation and almost identical means and standard deviations with the 10-item ISMI. Finally, the study focused exclusively on the participants' experienced self-stigma without examining its impact on their mental health outcomes, such as their quality of life.

## Conclusion

PWMI in Qatar reported different levels of perceived internalized stigma. An anti-stigma education program should take into consideration educating PWMIs and increasing social support.

## Data Availability Statement

After being de-identified and in compliance with the policies and procedures of all universities involved and HMC and Qatar National Research Fund, the datasets generated and/or analyzed during the current study are available from the corresponding author on reasonable request. Requests to access the datasets should be directed to vkehyaya@ucalgary.ca.

## Ethics Statement

The studies involving human participants were reviewed and approved by Hamad Medical Corporation - Qatar, Weill Cornell Medicine - Qatar, and University of Calgary - Canada. The patients/participants provided their written informed consent to participate in this study.

## Author Contributions

VK was involved in designing the study, data collection, data analysis, drafting, and critically reviewing the manuscript. SG and HA-A were involved in designing the study and critically reviewing the manuscript. ZM was involved in conducting the statistical analysis and writing up the statistical analysis and results sections of the manuscript. TM was the lead in data collection, data entry and ensuring data quality, and preparing the data presentation in the manuscript. All authors read and approved the final manuscript.

## Conflict of Interest

The authors declare that the research was conducted in the absence of any commercial or financial relationships that could be construed as a potential conflict of interest.

## Abbreviations

ADHD: Attention Deficit/Hyperactive Disorder
CI: Confidence Interval
DSM: Diagnostic and Statistical Manual of Mental Disorders
HMC: Hamad Medical Corporation
ICD: International Classification of Diseases
ISMI: Internalized Stigma Mental Illness
MHS: Mental Health Service
OR: Odds Ratio
NPRP: National Priorities Research Program
OCD: Obsessive Compulsive Disorder
PWMI: Person with Mental Illness
QR: Qatari Riyal
SD: Standard Deviation
SE: Standard Error
US: United States
RA: Research Assistant
USID: Unique Study Identification Numbers.
